# Complicated Diverticulitis: Age Distribution, Management and Burden on Health Care

**DOI:** 10.7759/cureus.34482

**Published:** 2023-02-01

**Authors:** Hassan Al-Saadi, Haider Abdulrasool, Elizabeth Murphy

**Affiliations:** 1 General Surgery Unit, Western Health, Melbourne, AUS; 2 General Surgery Unit, Lyell McEwin Hospital, Adelaide, AUS; 3 Colorectal Surgery Unit, Lyell McEwin Hospital, Adelaide, AUS

**Keywords:** distribution of complications of acute diverticulitis, length of stay of complicated diverticulitis, age distribution of complicated diverticulitis, complicated diverticulitis conservative management rate, complicated diverticulitis burden

## Abstract

Introduction: Acute diverticulitis represents a common surgical condition and one of the leading gastrointestinal causes of hospital admissions in Western societies. The aim of the study is to examine the distribution, management and cost to healthcare of complicated diverticulitis and compare those to uncomplicated diverticulitis.

Methods: The case-control study was performed for patients with acute diverticulitis in Lyell McEwin Hospital in Adelaide, South Australia. Data were collected for patients presented from January 2015 to December 2017. Consecutive patients with acute diverticulitis confirmed by computed tomography were included in the study. Patients recruited for the study were divided into two groups. Patients presenting with Hinchey Ia diverticulitis were classified as ‘uncomplicated diverticulitis’. Patients who presented with Hinchey Ib, II, III or IV diverticulitis were classified as ‘complicated diverticulitis’. The Hinchey classification was based on the radiological reports of CT scans.

Results: From 2015-2017, 116 cases were screened for the study, 10 of which were excluded due to not having CT diagnosis. A total of 106 consecutive cases of acute diverticulitis were recruited for the study. Forty-four cases had complicated diverticulitis. Sixty-two cases with uncomplicated diverticulitis were allocated as a control group. The distribution of cases spanned through all age groups. There were nine cases (20.9%) in the 30-39 age group in the complicated diverticulitis compared to eight cases (12.9%) in the uncomplicated group with odds ratio 1.7 (0.61-4.92). The mean length of stay of the complicated diverticulitis group was 7.74 days compared to 3.93 days of the uncomplicated group with a p value of 0.000235. Nine (20%) cases of the 44 complicated diverticulitis cases were managed operatively, while 35 (80%) of the complicated diverticulitis group and all of the uncomplicated (control) group were managed conservatively. Localized perforations were 24 cases (54.5%) of the complicated diverticulitis group and collections were 18 cases (40.8%). Those cases collectively represented the majority of the complicated group.

Conclusion: Complicated diverticulitis increases the length of stay significantly in acute diverticulitis cases that are requiring hospital admission despite conservative management in 80% of the cases. Younger age groups represent a significant percentage of both complicated and uncomplicated diverticulitis. In the study population, the percentage of the younger age group was higher in complicated diverticulitis compared to uncomplicated diverticulitis, although this increased risk did not reach statistical significance. This will need to be further investigated in future studies.

## Introduction

Diverticular disease is a range of signs and symptoms related to the presence of diverticula in the colon wall. These may include infection, perforation, bleeding, fistula, and occasionally obstruction due to chronic inflammation. It was first described in the mid-19th century and appears to be an unfortunate product of the Industrial Revolution and is thought to be due to changes in diet. The incidence has been reported to increase with age and has been largely on the rise in Western societies. Approximately 30% of those older than 60 years and roughly 60% to 80% of those older than 80 years may be affected. Only 10% to 20% of people with diverticular disease develop symptoms [1].

There are two classifications of diverticular disease, the clinical classification and the Hinchey classification. In clinical classification, diverticular disease is classified as follows: symptomatic uncomplicated disease, recurrent symptomatic disease, and complicated disease. Complicated disease includes hemorrhage, abscess, phlegmon, perforation, purulent and faecal peritonitis, stricture, fistula, and small-bowel obstruction due to post inflammatory adhesions. The modified Hinchey classification describes the clinical stages of perforated diverticular disease. These include stage I (pericolic abscess), stage IIa (distant abscess amenable to percutaneous drainage), stage IIb (complex abscess associated with/without fistula), stage III (generalized purulent peritonitis) and stage IV (fecal peritonitis) [2].

The incidence of diverticulitis is increasing. The number of inpatient hospitalizations is also rising. In the United States, acute diverticulitis admissions have increased by 26% from 1998 to 2005 [3]. The largest increase was in the younger population aged 18 to 44 years (82%). It was also reported that elective operations for diverticulitis have increased by 29% with the largest increase in patients aged 18 to 44 years (73%) [4].

Diverticular disease of the colon represents an important cause of inpatient hospital admissions and a significant contributor to health care costs in Western and industrialized societies [5,6]. In the United States, acute diverticulitis represents the third most common gastrointestinal illness that requires hospitalization, and it is the leading indication for elective colon resection [7-9].

The effect of patient age on the severity of acute diverticulitis has been studied. In the available literature, the results of these studies are conflicting. One study found that older age (> 70 years) is associated with severe disease [10]. Another study showed more severe disease in patients younger than 50 years [11]. Other studies found no correlation between severity of acute diverticulitis and age [12-14].

There are multiple modalities of management of acute diverticulitis. These include conservative management, radiological drainage procedures for diverticular collections and operative management. Conservative management has been recognized to be an effective management modality even in cases of complicated diverticulitis [15].

The aim of the study is to compare the age distribution, management and cost to health care of complicated diverticulitis to those of uncomplicated diverticulitis.

## Materials and methods

The case-control study was performed for patients with acute diverticulitis in Lyell McEwin Hospital in Adelaide, South Australia. Data were collected for consecutive patients presented from January 2015 to December 2017.

Patients with diverticular disease were identified through a diagnosis-specific code used in the hospital. Patients with acute diverticulitis confirmed by computed tomography were included in the study. Patients with diverticular bleeding, no CT confirmation and incidental diverticular disease on colonoscopy were excluded.

Patients recruited for the study were divided into two groups. Patients presenting with Hinchey Ia diverticulitis were classified as ‘uncomplicated diverticulitis’. Patients who presented with Hinchey Ib, II, III or IV diverticulitis were classified as ‘complicated diverticulitis’. The Hinchey classification was based on the radiological reports of CT scans.

Multiple parameters were collected for each patient. These included patient characteristics, number of attacks of diverticulitis, presence of immunosuppression, past history of complicated diverticulitis, computed tomography findings, length of stay in hospital and management of these patients. These parameters were extracted from patient records, laboratory and radiology records. Immunosuppression was defined as the presence of factors leading to the diminution of host defenses such as a concurrent history of immunosuppressant medication (such as chronic glucocorticoids, azathioprine, cyclosporine, methotrexate, tacrolimus, and anti-tumor necrosis factor agents), solid organ transplant, extra-colonic active malignant neoplasm, cytotoxic chemotherapy, and congenital or acquired immunodeficiency syndrome. The parameters of patients with complicated diverticulitis were compared to those of patients with uncomplicated diverticulitis as a control group.

Binary and continuous data were collected. Some continuous variables were either changed to binary data or classified in categorical data that are more relevant to clinical practice or more practical. This includes age group categories. Those parameters were compared between the two groups to investigate the distribution and the difference in disease burden to health care between the two groups. Data analysis was performed in form of 2x2 tables for binary data, with Chi square as the test of significance. For continuous data, comparison of means and t-test were used for analysis and test of significance.

## Results

From January 2015 to December 2017, 116 consecutive cases were screened for the study, 10 of which were excluded due to not having CT diagnosis. A total of 106 cases were recruited. Forty-four cases had complicated diverticulitis. Sixty-two cases with uncomplicated diverticulitis were allocated as a control group. The two groups were comparable in demographics and relevant characteristics (Table 1).

**Table 1:** Baseline characteristics of the study groups

	Complicated (Case) group	Uncomplicated (control) group
Frequency	44	62
Sex (%)		
Male	26 (59%)	32 (52%)
Female	18 (41%)	30 (48%)
Mean age (SD)	56.7 (17)	56.2(14.6)
First episode	23 (52.3%)	24 (38.7%)
Recurrent episode	21 (47.7%)	38 (61%)
Immune Suppression	3 (6.8%)	5 (8%)
Previous complicated diverticulitis	8 (18.2%)	11 (17.8%)

The age distribution of both groups was compared and reported (Table 2). The distribution of cases spanned through all age groups. There were nine cases (20.9%) in the 30-39 age group in the complicated diverticulitis compared to eight cases (12.9%) in the uncomplicated group with an odds ratio of 1.7 (0.61-4.92).

**Table 2:** Age distribution of study groups

Age group (years)	Complicated cases (%)	Uncomplicated cases (%)
30-39	9 (20.9%)	8 (12.9%)
40-49	8 (18.6%)	16 (25.8%)
50-59	8 (18.6%)	12 (19.4%)
60-69	6 (14%)	12 (19.4%)
70-79	8 (18.6)	11(17.7%)
80-89	4 (9.3%)	3 (4.8%)

The length of stay in hospitals can be used to reflect the cost on health care system. We found that the mean length of stay of the complicated diverticulitis group was 7.74 days compared to 3.93 days in the uncomplicated diverticulitis group. This difference in the length of stay was statistically significant with p value of 0.000235.

Nine (20%) cases of the 44 complicated diverticulitis cases required operative management, while 35 (80%) of the complicated diverticulitis group and all of the uncomplicated (control) group were managed conservatively (Table 3).

**Table 3:** Length of stay and management comparison ^1 ^Standard deviation

	Complicated (Case) group	Uncomplicated (control) group	Overall
Mean length of Stay in days (SD)^1^	7.74 (5.98)	3.97 (1.5)	5.49 (4.36)
Minimum	2	1	1
Maximum	34	10	34
Management (%)			
Operative	9 (20%)	0	9 (8%)
Conservative	35 (80%)	62 (100%)	97 (92%)

Table 4 details the different complications of acute diverticulitis in the study population with their corresponding length of stay and management. There were 24 cases (54.5%) of localized perforations and collections were 18 cases (40.8%). Those cases represented the majority of the complicated diverticulitis group.

**Table 4:** Comparison of mean length of stay and management in different types of complicated diverticulitis

	Abscess <3cm	Abscess>=3cm	Localized Perforation	Free Perforation	Total
Frequency (%)	5 (11.3%)	13 (29.5%)	24 (54.5 %)	2 (4.5%)	44
Management (%)					
Conservative	5 (11.3 %)	9 (20.4 %)	21 (47.7 %)	0	35 (79.5 %)
Operative	0	4 (9 %)	3 (6.8 %)	2 (4.5 %)	9 (20.5 %)
Length of stay in days (SD)					
Conservative	7 (3.7)	5.78 (3.27)	5.71 (2.8)	0	4.67 (2.35)
Operative	0	13.67 (1.5)	16.5 (9)	22 (17)	16.86 (8.76)
Total	7 (3.7)	7.75 (4.58)	6.65 (4.56)	22 (17)	

## Discussion

The observational study was conducted to compare the age distribution, management and length of stay between complicated diverticulitis and uncomplicated diverticulitis.

The two study groups were comparable in demographics and relevant characteristics. This is an important aspect to minimize the effect of confounding.

The age distribution was found to span across all the age categories, starting at a younger age than previously expected. The percentage of cases in the 30-39 age group was higher (20.9%) in the complicated group compared to the uncomplicated group (12.9%). The odds ratio was 1.7 which indicates that there is an increased risk of complicated diverticulitis in the study population with the younger age group. However, this correlation did not reach statistical significance. The study also highlighted that diverticulitis cases are significant in the younger population compared to what was previously thought. The percentage of the younger population requiring hospital admission for acute diverticulitis is also significant compared to the overall age distribution.

The mean length of stay was significantly longer and almost double in the complicated group compared to the uncomplicated. This is despite the fact that the majority of complicated diverticulitis cases are being managed conservatively. The length of stay in hospital is a major contributor to health care cost and burden to the hospital system. With current increase in population and difficulties in expanding hospital capacities due to funding issues, there is an ongoing rise in inpatient bed demand.

The study limitations can be attributed to a small sample size which limits the generalizability of the findings. This would be a limiting factor to reach statistical significance in the identified correlations. Another limitation was limited number of cases that underwent radiological drainage procedure during the three years of the study. All these cases failed this modality of management and proceeded to operative management.

## Conclusions

Younger age groups represent a significant percentage of both complicated and uncomplicated diverticulitis. In the study population the percentage of the younger age group (30-39 years) was higher in the complicated diverticulitis group compared to the uncomplicated diverticulitis group, although this increased risk did not reach statistical significance likely due to limitation in the sample size of the study. This will need to be further investigated in future studies with larger study population.

Complicated diverticulitis was found to significantly increase the length of stay in hospital. This translates directly to increase in cost to the health care system and the rising demand for inpatient beds. Localized perforation and collections represent the majority of the cases of complicated diverticulitis and more severe complication are less common. Conservative management of acute diverticulitis is the main modality of management. This is clearly evident as all the cases of uncomplicated diverticulitis and 80% of the cases of complicated diverticulitis are managed conservatively.

## References

[1] Sheth AA, Longo W, Floch MH (2008). Diverticular disease and diverticulitis. Am J Gastroenterol.

[2] Köhler L, Sauerland S, Neugebauer E (1999). Diagnosis and treatment of diverticular disease: results of a consensus development conference. The Scientific Committee of the European Association for Endoscopic Surgery. Surg Endosc.

[3] Etzioni DA, Mack TM, Beart RW Jr, Kaiser AM (2009). Diverticulitis in the United States: 1998-2005: changing patterns of disease and treatment. Ann Surg.

[4] Pemberton JH, Friedman LS, Grover S (2023). Colonic diverticulosis and diverticular disease: epidemiology, risk factors, and pathogenesis. UpToDate.

[5] Everhart JE, Ruhl CE (2009). Burden of digestive diseases in the United States part II: lower gastrointestinal diseases. Gastroenterology.

[6] Shaheen NJ, Hansen RA, Morgan DR (2006). The burden of gastrointestinal and liver diseases, 2006. Am J Gastroenterol.

[7] Peery AF, Dellon ES, Lund J (2012). Burden of gastrointestinal disease in the United States: 2012 update. Gastroenterology.

[8] Anaya DA, Flum DR (2005). Risk of emergency colectomy and colostomy in patients with diverticular disease. Arch Surg.

[9] Pemberton JH, Weiser M, Raghavendran K (2023). Acute colonic diverticulitis: medical management. UpToDate.

[10] Ambrosetti P, Robert JH, Witzig JA, Mirescu D, Mathey P, Borst F, Rohner A (1994). Acute left colonic diverticulitis: a prospective analysis of 226 consecutive cases. Surgery.

[11] Hall JF, Roberts PL, Ricciardi R (2010). Colonic diverticulitis: does age predict severity of disease on CT imaging?. Dis Colon Rectum.

[12] Ritz JP, Lehmann KS, Stroux A, Buhr HJ, Holmer C (2011). Sigmoid diverticulitis in young patients--a more aggressive disease than in older patients?. J Gastrointest Surg.

[13] Lopez-Borao J, Kreisler E, Millan M (2012). Impact of age on recurrence and severity of left colonic diverticulitis. Colorectal Dis.

[14] Lorimer JW, Doumit G (2007). Comorbidity is a major determinant of severity in acute diverticulitis. Am J Surg.

[15] Dharmarajan S, Hunt SR, Birnbaum EH, Fleshman JW, Mutch MG (2011). The efficacy of nonoperative management of acute complicated diverticulitis. Dis Colon Rectum.

